# Assessing Interpersonal Proximity Evaluation in the COVID-19 Era: Evidence From the Affective Priming Task

**DOI:** 10.3389/fpsyg.2022.901730

**Published:** 2022-06-16

**Authors:** Elisa Scerrati, Stefania D'Ascenzo, Roberto Nicoletti, Caterina Villani, Luisa Lugli

**Affiliations:** ^1^Department of Biomedical, Metabolic and Neural Sciences, University of Modena and Reggio Emilia, Reggio Emilia, Italy; ^2^Department of Philosophy and Communication, University of Bologna, Bologna, Italy

**Keywords:** pandemic, social distancing, prime images, target words, affective priming, interpersonal proximity

## Abstract

Social proximity has since ever been evaluated as positive. However, the outbreak of the COVID-19 pandemic has dramatically reduced our social relations to avoid spreading the contagion. The present study aims to investigate people's current assessment of social proximity by using an affective priming paradigm (APP). We hypothesized that if our evaluation of social proximity is positive, then words with positive valence (e.g., relaxed) should be processed faster when preceded by images of social proximity than social distancing. On the contrary, if our evaluation of social proximity is turning negative, then words with a negative valence (e.g., sad) should be processed faster when preceded by images of social proximity than social distancing. To this end, we presented participants with prime images showing line drawings representing humans in situations of proximity or distancing and asked them to evaluate the valence (i.e., positive or negative) of a subsequent target word. In a follow-up session, the same participants evaluated the prime images as being positively or negatively valenced. Results showed that a large subset of participants who rated the prime images of social proximity as positive also processed positive words faster when these were preceded by images of social proximity than social distancing. Conversely, a smaller subset of participants who rated the prime images of social proximity as less positive processed negative words faster when these were preceded by images of social proximity than social distancing. These results suggest individual differences in the assessment of social proximity likely driven by the pandemic.

## Introduction

Social proximity has since ever been evaluated as positive (e.g., Beckes and Coan, 2011). According to an evolutionary perspective, it is the primary way in which primates establish and maintain social bonds (e.g., Dunbar, 2010). Research has shown that health and wellbeing are improved by close social relationships and rich social networks (e.g., Gallagher and Vella-Brodick, 2008; Cohen and Janicki-Deverts, 2009). However, the outbreak of the COVID-19 pandemic has dramatically impacted our social relations by reducing interpersonal proximity because potentially dangerous (e.g., Xie et al., 2020; Xu and Cheng, 2021). Recent studies have shown that social distancing (i.e., avoiding contact with others) imposed by the pandemic influenced our cognitive abilities (e.g., Santangelo et al., 2021; D'Ascenzo et al., 2022) as well as our social interactions (e.g., Cartaud et al., 2020), mental health (e.g., Maggi et al., 2021), and wellbeing (e.g., De Pue et al., 2021). In addition, it has been shown that the concept of COVID-19 is strongly related to the concept of fear, and it is associated with social scenarios (Mazzuca et al., 2021). However, it has also been pointed out that collective threats (such as COVID-19), rather than pushing us toward a defensive avoidance behavior, end up making us seek even more physical and psychological closeness (Dezecache et al., 2020). Indeed, it is suggested that threats generate stress that we tend to handle by providing and requiring social support. The reinforcement of social inclination may be especially true for disease, such as COVID-19 that are largely invisible and remain asymptomatic in a large part of the population.

The present study aims at investigating people's current assessment of social proximity by using an affective priming paradigm (APP). By “priming effect,” we commonly refer to the influence that one stimulus exerts on how people respond to a subsequent stimulus (e.g., Meyer and Schvaneveldt, 1971; Collins and Loftus, 1975). For example, the word *cat* is recognized faster following the word *dog* than the word *bread*. More generally, priming occurs whenever exposure to one thing affects an individual's subsequent behavior or performance on a given task (e.g., Scerrati et al., 2015, 2017, 2021). The affective priming effect (henceforth APE) refers to the finding of faster and more accurate responses to valenced (i.e., positive or negative) target stimuli that are preceded by affectively congruent (i.e., positive-positive and negative-negative) rather than affectively incongruent (i.e., positive-negative and negative-positive) prime stimuli (see Klauer and Musch, 2003 for a review). In a typical APP, participants first evaluate a series of object words (e.g., flowers) as good or bad in meaning. The words categorized fastest as good and bad then serve as prime stimuli in a second phase where the same people evaluate a series of adjective targets (e.g., delightful) preceded by the object words. APE has been shown with different combinations of prime-target, such as word-word (e.g., Fazio et al., 1986; Bargh et al., 1996; Klauer et al., 1997; Hermans et al., 2001) as well as picture-word (e.g., Spruyt et al., 2002, 2018), and picture-picture (e.g., Hermans et al., 1994; Lugli et al., 2014), with either evaluative (e.g., Fazio et al., 1986; Hermans et al., 1994, 2001; Bargh et al., 1996; Klauer et al., 1997; Wu et al., 2021), pronunciation (e.g., De Houwer et al., 1998), and lexical decision (e.g., Wentura, 2000) tasks. Most importantly, APP has been used as an indirect measure of other attitudes, such as food liking (e.g., Tzavella et al., 2020). In particular, Tzavella et al. (2020) asked participants to respond to the valence (positive-negative) of target words (e.g., smile) after briefly seeing prime images of healthy (e.g., watermelon) or unhealthy (e.g., crisps) foods that were then masked. Results showed robust priming effects for both healthy and unhealthy foods, providing strong evidence that the APP can be used as an indirect measure of other attitudes (i.e., food liking).

The present study aims at testing social proximity evaluation by using the picture-word evaluative APP as an indirect measure of interpersonal proximity behaviors. We specifically tested whether our implicit evaluation of the interpersonal proximity/distance affects the processing of positive/negative adjective words. Bearing in mind that close proximity and interaction are essential to the human brain (Beckes and Coan, 2011) since they allow primates to establish and maintain social bonds (e.g., Dunbar, 2010), but also considering the self-defensive functions associated with the regulation of the interpersonal space (Coello and Cartaud, 2021) and that interpersonal proximity has recently become potentially dangerous because of the pandemic (e.g., Xie et al., 2020; Xu and Cheng, 2021), we hypothesized that if our evaluation of social proximity is still positive despite the pandemic, then words with positive valence (e.g., relaxed) should be processed faster when preceded by images of social proximity than social distancing. On the contrary, if our evaluation of social proximity is turning negative because of the pandemic, then words with a negative valence (e.g., sad) should be processed faster when preceded by images of social proximity than social distancing. To this end, we presented participants with prime images showing line drawings representing humans in situations of proximity or distancing and asked them to evaluate the valence (i.e., positive or negative) of a subsequent target word. In a follow-up session, the same participants evaluated the prime images as being positively or negatively valenced.

## Methods

### Participants

To note, in the context of the current experiment, APE is the difference between incongruent (i.e., proximity-negative and distancing-positive) and congruent (i.e., proximity-positive and distancing-negative) prime-target pairs. Therefore, APE is a function of Congruence (congruent or incongruent). We calculated the sample size required to achieve 80% power to detect a significant main effect of *Congruence* (congruent or incongruent) with G^*^power 3.1 (Faul et al., 2007). With an effect size of *f* = 0.25 (Cohen, 1988), the power calculation gave a recommended sample size of at least 34 participants. In total, 40 students (31 women; mean age: 21 years old; and SD: 5 years) from the University of Bologna took part in the experiment. They all reported themselves as being right-handed. They all had normal or corrected-to-normal vision and were naive as to the purpose of the experiment. They all served as unpaid volunteers. The experiment was conducted in accordance with the ethical standards laid down in the Declaration of Helsinki and fulfilled the ethical standard procedure recommended by the Italian Association of Psychology (AIP). The procedures were approved by the ethics committee of the University of Bologna. All participants gave their written informed consent prior to participate in the study.

### Apparatus and Stimuli

We used the online platform Gorilla Experiment Builder (www.gorilla.sc) to create and host our experiment (Anwyl-Irvine et al., 2020; for a critical overview of the platform see Scerrati et al., 2022). Automated procedures ensured that participants were all using a desktop computer and automatically rejected participants who took more than 2 h to complete the task. To minimize potential distractions, participants were invited to carry out the experiment in a quiet place and to avoid the manipulation of any object throughout the task. In addition, before starting, participants were asked to close background apps, software, and all browser windows except for that of the experiment.

**Figure 1:**
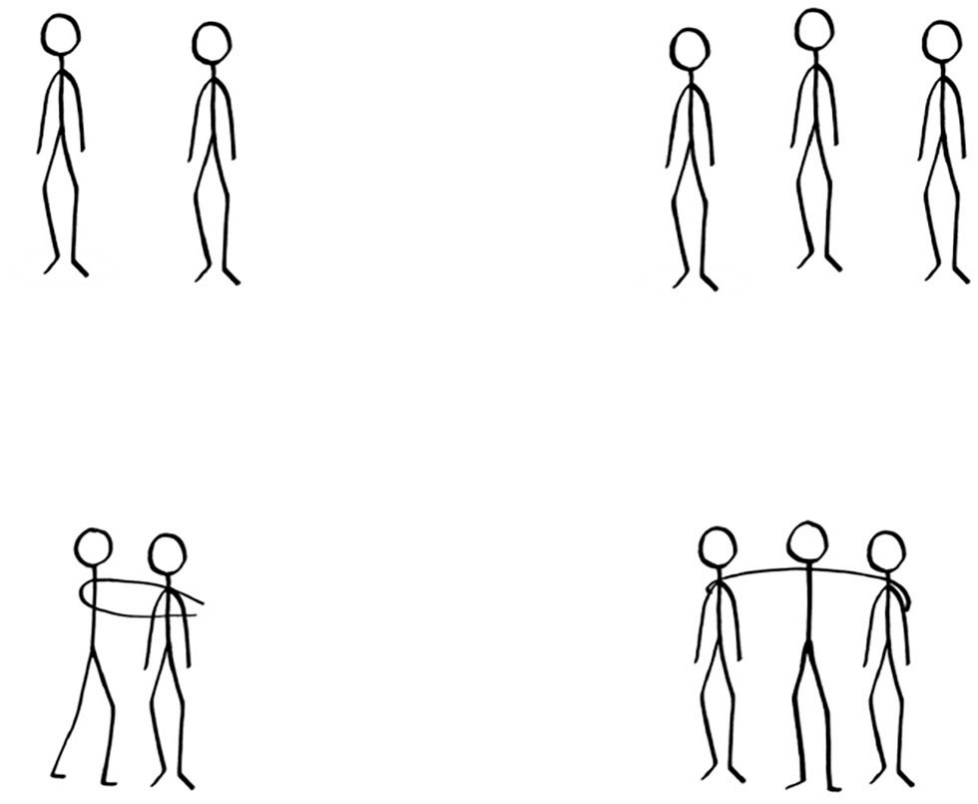
A schematic representation of the prime images in the distancing (top panel) and proximity (bottom panel) prime conditions with 2 (leftward panel) and 3 (rightward panel) stick figures. Note that elements are not drawn to scale.

Four prime images were selected from the Interpersonal Relations Picture System (Fuchs et al., 2018), a database encompassing picture stimuli of interpersonal situations. Two images represented humans in situations of proximity and two images represented humans in situations of distancing (as shown in Figure 1).

Sixteen target words were selected from the Italian database of affective norms by Montefinese et al. (2014), which also provides objective (e.g., word frequency) and subjective (e.g., familiarity) psycholinguistic indexes. Eight were positive (*bold, confident, elegant, hopeful, lively, lucky, protected, and relaxed*) and 8 were negative (*confused, depressed, discouraged, frustrated, insecure, nervous, sad, and troubled*) emotion-label adjectives (Wu et al., 2021). They differed in valence and dominance^1^ and were matched for arousal, number of letters, word frequency,^2^ familiarity, imageability, and concreteness (as shown in Appendix).

### Procedure

Participants' task was to judge whether the target word had a positive or negative meaning as rapidly and accurately as possible (e.g., Fazio et al., 1986; Wu et al., 2021) by pressing a left key (T or Y of a QWERTY keyboard depending on whether it had or not the numeric pad on the right) with their left index finger to indicate a negative target and a right key (I or O of a QWERTY keyboard depending on whether it had or not the numeric pad on the right) with their right index finger to indicate a positive target. This response mapping assignment was counterbalanced across participants.

Each trial started with a black fixation cross (0.5 × 0.5 cm) that appeared on a white background at the center of the screen for 500 ms. Subsequently, the prime (8 cm in height and ranging in width from 4 to 8 cm) was displayed centrally for 200 ms followed by a 50 ms blank screen for a total of 250 ms Stimulus-Onset Asyncrony (SOA). At this point, the target appeared centrally on-screen in Open-sans 36 and was visible for 2,000 ms or until the participant's response. The intertrial interval was 2,500 ms.

Participants performed 16 practice trials with different target words from the experimental ones followed by two blocks of 64 trials each, for a total of 144 trials per participant. There were 32 trials for each condition (i.e., proximity-positive; proximity-negative; distancing-positive; and distancing-negative). The order of trials within each block was randomly determined. Blocks were separated by a self-paced interval and the experiment lasted ≈10 min.

## Results

### Statistical Analysis

In this study, one female participant was excluded from the analyses due to a large number of errors (error rate: 32%), which was over 10 times the group mean (2.82%). Therefore, 39 participants remained for the analyses. Practice trials, omissions (1.56%), errors (3.38%), and response times (RTs) faster/slower than the overall participant's mean minus/plus 2 SD (4.73%) were excluded from the analysis on RTs.

We defined congruent trials as those in which positive target words were preceded by proximity prime images and negative target words were preceded by distancing prime images and incongruent trials as those in which positive target words were preceded by distancing prime images and negative target words were preceded by proximity prime images. We calculated the APE by subtracting the mean reaction time on congruent trials from the mean reaction time on incongruent trials.

#### ANOVA

Two repeated-measures ANOVAs with *Congruence* (congruent or incongruent) as the within-subject factor were conducted separately on RTs and percentage errors (PEs).

Neither the analysis on RT nor that on PEs revealed a significant main effect of *Congruence, F*_*s*_ <1 (RT: M_congruent_ = 724 ms, SD_congruent_ = 125 ms; *M*_incongruent_ = 726 ms, SD_incongruent_ = 119 ms; PEs: *M*_congruent_ = 2.6%, SD_congruent_ = 2.4%; *M*_incongruent_ = 2.5%, and SD_incongruent_ = 3.1%).

### Additional Analyses

#### Bayesian Analysis

Based on the procedure of null hypothesis significance testing (NHST), the null hypothesis can never be accepted, one just fails to reject it (e.g., Lakens et al., 2020). Therefore, we performed a Bayesian *t*-test aimed at testing whether the absence of a significant difference between the congruent and incongruent conditions for both RTs and PEs can be taken as evidence in favor of the null hypothesis. The Bayesian *t*-test (Rouder et al., 2009) was performed in R 3.6.1 (R Core Team, 2016) with the “Bayes factor” library, using the default JZS prior and was aimed at comparing the probability of the null and the alternate hypothesis (H_o_ and H_1_, respectively) relative to the absence of a significant difference between the congruent and incongruent conditions. We found that the Bayes factor (BF_o1_) expressing the probability of the data given H_o_ (i.e., no difference) relative to H_1_ (i.e., difference) was BF_o1_ = 5.3 and BF_o1_ = 5.6 for RTs and PEs, respectively (Raftery, 1999; Wagenmakers, 2007). That is, H_o_ is 5 times more likely than H_1_ for both RTs and PEs. This result further suggests the absence of a significant effect of congruence.

#### Cluster Analysis

**Figure 2:**
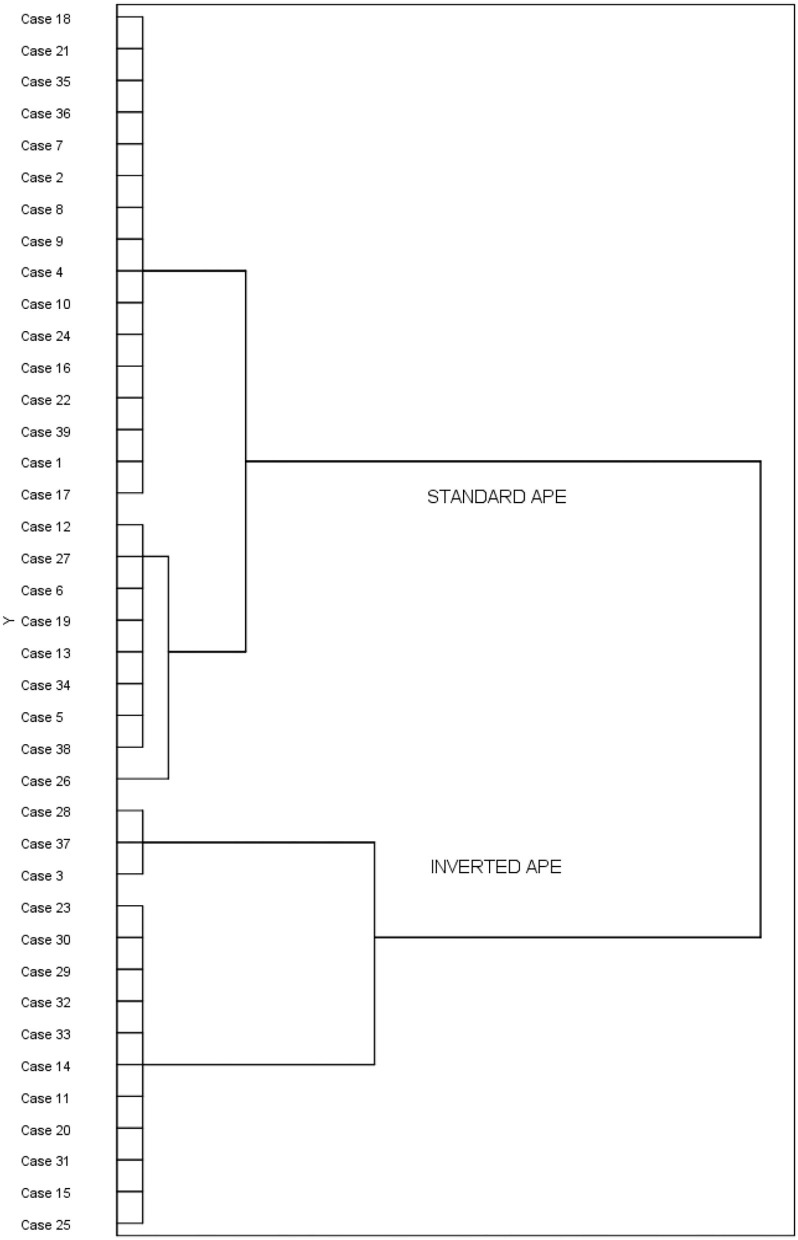
The dendrogram of hierarchical cluster analysis. Cases represent participants.

Since our hypothesis was two-tailed as interpersonal proximity conveyed by the prime images could be taken as either good for establishing and maintaining social bonds, or bad because potentially dangerous, we proceeded with a closer inspection of our data. To better visualize the data, we conducted a hierarchical agglomerative cluster analysis using Ward's method (Ward, 1963) with squared Euclidean distances as a measure of similarity. This procedure allowed us to create an empirical metric of how participants grouped together for the size of the APE. A 2-cluster solution emerged. The dendrogram reported in Figure 2 shows that a large subset of participants (25 out of 39) was included in the first cluster encompassing people who showed a standard APE, that is, faster congruent (i.e., proximity-positive and distancing-negative) than incongruent (i.e., proximity-negative and distancing-positive) trials, whereas a smaller subset of participants (14 out of 39) was included in the second cluster encompassing people who showed an inverted APE, i.e., faster incongruent (i.e., proximity-negative and distancing-positive) than congruent (i.e., proximity-positive and distancing-negative) trials (see Haaf and Rouder, 2019 for a recent discussion on inverted effects).

#### Mixed ANOVA

Given this pattern of results, we performed a mixed ANOVA with *Congruence* (congruent or incongruent) as the within-subject factor and *Group* (standard APE and inverted APE) as the between-subject factor, separately for each dependent variable (RTs and PEs).

##### Response Times

No main effect was significant, *F*_*s*_ < 1.87 *p*_*s*_ > 0.180. The interaction between *Congruence* and *Group* was significant, *F*(1,32) = 49.03, *p* < 0.001, $n_{p}^{2}$ = 0.57, indicating that incongruent trials (M: 715 ms, SE: 23.9) were significantly slower than congruent trials (M: 699 ms, SE: 24.4) in the standard APE group, whereas incongruent trials (M: 746 ms, SE: 31.9) were significantly faster than congruent trials (M: 769 ms, SE: 32.6) in the inverted APE group.

Therefore, we unpacked *Congruence* and performed the Bonferroni-corrected paired-sample *T*-tests aimed at comparing the effect of each kind of *Prime* (proximity and distancing) on either type of *Target* (positive and negative) for each *Group* (standard APE and inverted APE) separately. For the standard APE group, words were evaluated as positive 22 ms faster when preceded by proximity rather than distancing prime images, *t*(24) = 4.47, *p* < 0.001, whereas a small and non-significant advantage of 7 ms was observed for the processing of negative words when these were preceded by distancing rather than proximity images, *t*(24) = 1.48, *p* = 0.151. For the inverted APE group, words were evaluated as negative 27 ms faster when preceded by proximity than distancing images, *t*(13) = 3.48, *p* = 0.004, whereas a non-significant advantage of 17 ms was observed for the processing of positive words when these were preceded by distancing rather than proximity prime images, *t*(13) = 1.60, *p* = 0.133 (see Table 1 for details).

**Table 1:** Mean response times in milliseconds (with standard deviations (SDs) in parenthesis) as a function of kind of prime (proximity and distancing) and type of target (positive and negative) for each group (standard affective priming effect (APE) and inverted APE) separately.

	**Standard APE group (*****N*** **=** **25)**		**Inverted APE group (*****N*** **=** **14)**	
**Prime image**	**Target word**		**Target word**	
	**Positive**	**Negative**	**Positive**	**Negative**
Proximity	689 (111)	719 (105)	765 (145)	744 (124)
Distancing	711 (121)	712 (111)	748 (142)	771 (145)

##### Percentage Errors

Neither the main effects nor their interaction was significant (*F*_*s*_ < 1).

#### Follow-Up Questionnaire

To shed further light on these results, we conducted a follow-up questionnaire aimed at examining how participants belonging to the two groups (standard APE and inverted APE) evaluated the prime images. The questionnaire was administered online through Google forms 2 months after the main experiment. The same 39 participants who took part in the main experiment were asked to evaluate the behavior of the stick figures depicted in the prime images as being positive or negative on a 5-point Likert scale (1 = definitely positive, 5 = definitely negative).

In total, 34 out of the 39 original participants completed the questionnaire, 22 showing the standard APE, and 12 showing the inverted APE.

**Figure 3:**
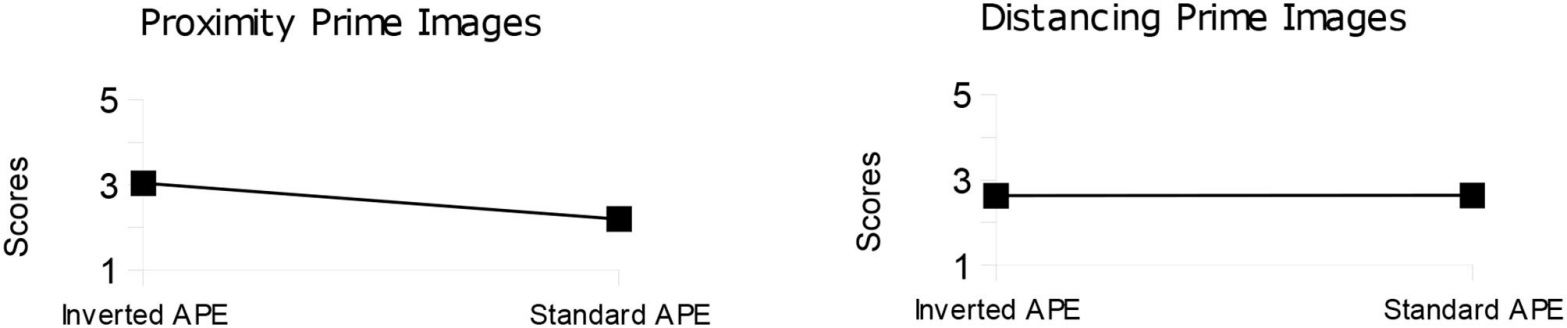
Mean scores for proximity (leftward panel) and distancing (rightward panel) prime images from participants showing the inverted and standard APE. Scores range from 1 (definitely positive) to 5 (definitely negative).

Independent-sample *t*-tests were conducted to compare participants' scores on the follow-up questionnaire concerning the prime images. Participants with the standard APE rated the proximity prime images as significantly more positive than participants showing the inverted APE, *t*(32) = 2.24, *p* = 0.032, whereas no difference between the two groups emerged when evaluating distancing prime images, *t*(32) = 0.029, *p* = 0.977 (as shown in Figure 3).

## Discussion

The COVID-19 pandemic forced us to reduce interpersonal proximity to avoid spreading the contagion. The present study tested people's current evaluation of social proximity by using a picture-word evaluative APP.

Results showed that people who rated images of interpersonal proximity as positive were also facilitated in recognizing words as positive in meaning when these were preceded by proximity rather than distancing images. Conversely, people who rated the prime images of social proximity as less positive were facilitated in recognizing words as negative in meaning when these were preceded by proximity rather than distancing images. In other words, for a large group of participants, social proximity images evoked something positive, whereas for a smaller subset they evoked something negative. Taken together, these results suggest sharp individual differences in the current assessment of social proximity and confirm the psychological roots of individual differences in the evaluation of social proximity/distancing (Xie et al., 2020; Xu and Cheng, 2021). Of course, observed individual differences may stem from a number of reasons inherent to the individuals involved in the study, such as their own perceived vulnerability to disease and their inclination toward risk perception, or their morality and adherence to social norms, and still their loneliness and need for affiliation or physical contact. However, given we did not measure any potential dispositional or situational factor that may be responsible for the observed differences in the sample, we may only speculate on the underlying sources of these differences.

Furthermore, it is worth considering that although taken from a validated dataset, the line drawings used in the current study do not carry any relevant cue in the context of the pandemic (such as wearing protective equipment or not, being infectious or not, or the interactants' social identity). This could be related to the lack of a consistent pattern across participants and the emergence of individual differences. Indeed, a large body of recent evidence suggests that contextual information (i.e., mask-wearing and social categories) and risk perception are key components in the regulation of proxemic behavior during the pandemic (e.g., Cartaud et al., 2020; Fini et al., 2021; Iachini et al., 2021; Lisi et al., 2021; Kroczek et al., 2022; Villani et al., manuscript submitted for publication). In other words, the evaluation of social proximity might have become more context-sensitive after the COVID-19 outbreak compared with the past, which might explain why we observed no unique, clear direction of the APE.

It is worth highlighting that in the follow-up questionnaire, distancing primes were evaluated as neither positive nor negative by either group of participants (standard APE and inverted APE: as shown in Figure 3, rightward panel). This result likely reflects the way we currently conceptualize distancing, that is, as neither natural nor deviant from an affective point of view (e.g., Szczurek et al., 2012).

In line with previous studies, our findings demonstrate affective priming even with this modified version of the APP, confirming that APP can be used as an implicit measure of cognitive attitudes (e.g., Tzavella et al., 2020).

Furthermore, our results suggest an automatic encoding of affective information even if it is implicit and not task-relevant as that conveyed by our prime images, thus strengthening the *effect primacy hypothesis* (Murphy and Zajonc, 1993; De Houwer et al., 2002; see Klauer and Musch, 2003 for a review), which assumes that the processing of affective information is prioritized over other types of information and its retrieval precedes the retrieval of descriptive or conceptual information in memory.

In conclusion, our results suggest prominent individual differences in the assessment of social proximity likely driven by the pandemic. Future studies may build on our findings and enlarge the sample size to investigate the specific underlying reasons (e.g., perceived vulnerability to disease and risk perception, morality and adherence to social norms, loneliness and need for affiliation, and physical contact) driving the observed individual differences in the assessment of social proximity in problematic circumstances.

## Data Availability Statement

The original contributions presented in the study are included in the article/Supplementary Material, further inquiries can be directed to the corresponding author/s.

## Ethics Statement

The studies involving human participants were reviewed and approved by Ethics Committee of the University of Bologna. The patients/participants provided their written informed consent to participate in this study.

## Author Contributions

ES: conceptualization, methodology, software, data collection, data analyses, and writing—original draft preparation. SD'A: conceptualization, methodology, data analyses, and writing—reviewing and editing. RN: conceptualization, methodology, and supervision. CV: conceptualization, methodology, and writing—reviewing and editing. LL: conceptualization, methodology, writing—reviewing and editing, and supervision. All authors contributed to the article and approved the submitted version.

## Conflict of Interest

The authors declare that the research was conducted in the absence of any commercial or financial relationships that could be construed as a potential conflict of interest.

## Publisher's Note

All claims expressed in this article are solely those of the authors and do not necessarily represent those of their affiliated organizations, or those of the publisher, the editors and the reviewers. Any product that may be evaluated in this article, or claim that may be made by its manufacturer, is not guaranteed or endorsed by the publisher.

## Footnotes

^1^The relationship between valence (the pleasantness of a stimulus) and dominance (the degree of control exerted by a stimulus) is linear (i.e., words with a positive value have high dominance values, whereas words with a negative value have low dominance values). That is why the two word classes (positive/negative) show a significant difference as for dominance values (Warriner et al., 2013).

^2^Word frequency as retrieved from Colfis (Bertinetto et al., 1995) was used.

## Appendix

**Table:** Affective norms and psycholinguistic indexes retrieved from Montefinese et al. (2014).

**Index**	**Positive**	**Negative**	***p* value**
Valence	7,5	2,4	<0.001
Arousal	5,6	6	0.387
Dominance	5,9	3,9	<0.001
Letters	8,1	8,3	0.768
Frequency	70,1	83	0.709
Familiarity	6,5	6,6	0.808
Imageability	6,2	6,2	1
Concreteness	4,5	4,9	0.288

*Values concern the selection of the target words used*.

## References

[B1] Anwyl-Irvine A. L. Massonnié J. Flitton A. Kirkham N. Evershed J. K. (2020). Gorilla in our midst: an online behavioral experiment builder. Behav. Res. Methods 52, 388–407. 10.3758/s13428-019-01237-x 31016684 PMC7005094

[B2] Bargh J. A. Chaiken S. Raymond P. Hymes C. (1996). The automatic evaluation effect: unconditional automatic attitude activation with a pronunciation task. J. Exp. Soc. Psychol. 32, 104–128. 10.1006/jesp.1996.0005

[B3] Beckes L. Coan J. A. (2011). Social baseline theory: the role of social proximity in emotion and economy of action. Soc. Personal. Psychol. Compass 5, 976–988. 10.1111/j.1751-9004.2011.00400.x

[B4] Bertinetto P. M. Burani C. Laudanna A. Marconi L. Ratti D. Rolando C.. (1995). CoLFIS (Corpus e Lessico di Frequenza dell'Italiano Scritto)[Corpus and Frequency Lexicon of Written Italian]. Rome: Institute of Cognitive Sciences and Technologies.

[B5] Cartaud A. Quesque F. Coello Y. (2020). Wearing a face mask against COVID-19 results in a reduction of social distancing. PLoS ONE 15:e0243023. 10.1371/journal.pone.0243023 33284812 PMC7721169

[B6] Coello Y. Cartaud A. (2021). The interrelation between peripersonal action space and interpersonal social space: psychophysiological evidence and clinical implications. Front. Hum. Neurosci. 15:636124. 10.3389/fnhum.2021.636124 33732124 PMC7959827

[B7] Cohen J. (1988). Statistical power analysis for the behavioral sciences. Hillsdale (NJ): Lawrence Erlbaum Associates.

[B8] Cohen S. Janicki-Deverts D. (2009). Can we improve our physical health by altering our social networks? Perspect. Psychol. Sci. 4, 375–378. 10.1111/j.1745-6924.2009.01141.x 20161087 PMC2744289

[B9] Collins A. M. Loftus E. F. (1975). A spreading-activation theory of semantic processing. Psychol. Rev. 82, 407. 10.1037/0033-295X.82.6.407

[B10] D'Ascenzo S. Scerrati E. Villani C. Galatolo R. Lugli L. Nicoletti R. (2022). Does social distancing affect the processing of brand logos? Brain Behav. 12, 2501. 10.1002/brb3.2501 35212187 PMC8933757

[B11] De Houwer J. Hermans D. Eelen P. (1998). Affective and identity priming with episodically associated stimuli. Cogn. Emot. 12, 145–169. 10.1080/026999398379691

[B12] De Houwer J. Hermans D. Rothermund K. Wentura D. (2002). Affective priming of semantic categorisation responses. Cogn. Emot. 16, 643–666. 10.1080/0269993014300041929883263

[B13] De Pue S. Gillebert C. Dierckx E. M.-A. Vanderhasselt R. De Raedt E. Van den Bussche (2021). The impact of the COVID-19 pandemic on wellbeing and cognitive functioning of older adults. Sci. Rep. 11, 4636. 10.1038/s41598-021-84127-7 33633303 PMC7907111

[B14] Dezecache G. Frith C. D. Deroy O. (2020). Pandemics and the great evolutionary mismatch. Curr. Biol. 30, R417–R419. 10.1016/j.cub.2020.04.010 32428465 PMC7233247

[B15] Dunbar R. I. (2010). The social role of touch in humans and primates: behavioural function and neurobiological mechanisms. Neurosci. Biobehav. Rev. 34, 260–268. 10.1016/j.neubiorev.2008.07.001 18662717

[B16] Faul F. Erdfelder E. Lang A. G. Buchner A. (2007). G^*^ Power 3: a flexible statistical power analysis program for the social, behavioral, and biomedical sciences. Behav. Res. Methods 39, 175–191. 10.3758/BF03193146 17695343

[B17] Fazio R. H. Sanbonmatsu D. M. Powell M. C. Kardes F. R. (1986). On the automatic activation of attitudes. J. Pers. Soc. Psychol. 50, 229. 10.1037/0022-3514.50.2.229 3701576

[B18] Fini C. Tummolini L. Borghi A. M. (2021). Contextual modulation of preferred social distance during the Covid-19 pandemic. Sci. Rep. 11, 1–11. 10.1038/s41598-021-02905-9 34887441 PMC8660879

[B19] Fuchs S. Bohleber L. M. Ernst J. Soguel-dit-Piquard J. Boeker H. Richter A. (2018). One look is worth a thousand words: new picture stimuli of interpersonal situations. Soc. Neurosci. 13, 346–354. 10.1080/17470919.2017.1327457 28475471

[B20] Gallagher E. N. Vella-Brodick D. A. (2008). Social support and emotional intelligence as predictors of subjective well-being. Pers. Individ. Dif. 44, 1551–1561. 10.1016/j.paid.2008.01.011

[B21] Haaf J. M. Rouder J. N. (2019). Some do and some don't? Accounting for variability of individual difference structures. Psychon. Bull. Rev. 26, 772–789. 10.3758/s13423-018-1522-x 30251148

[B22] Hermans D. De Houwer J. Eelen P. (2001). A time course analysis of the affective priming effect. Cogn. Emot. 15, 143–165. 10.1080/02699930125768

[B23] Hermans D. Houwer J. D. Eelen P. (1994). The affective priming effect: automatic activation of evaluative information in memory. Cogn. Emot. 8, 515–533. 10.1080/02699939408408957

[B24] Iachini T. Frassinetti F. Ruotolo F. Sbordone F. L. Ferrara A. Arioli M.. (2021). Social distance during the COVID-19 pandemic reflects perceived rather than actual risk. Int. J. Environ. Res. Public Health 18, 5504. 10.3390/ijerph18115504 34063754 PMC8196577

[B25] Klauer K. C. Musch J. (2003). “Affective priming: findings and theories,” in The Psychology of Evaluation: Affective Processes in Cognition and Emotion, eds J. Musch, and K. C. Klaue (Mahwah, NJ: Lawrence Erlbaum Associates Publishers), 7–49.

[B26] Klauer K. C. Rossnagel C. Musch J. (1997). List-context effects in evaluative priming. J. Exp. Psychol. Learn. Mem. Cogn. 23, 246. 10.1037/0278-7393.23.1.246 9028030

[B27] Kroczek L. O. Böhme S. Mühlberger A. (2022). Face masks reduce interpersonal distance in virtual reality. Sci. Rep. 12, 1–10. 10.1038/s41598-022-06086-x 35140279 PMC8828850

[B28] Lakens D. McLatchie N. Isager P. M. Scheel A. M. Dienes Z. (2020). Improving inferences about null effects with Bayes factors and equivalence tests. J. Gerontol.: Ser. B 75, 45–57. 10.1093/geronb/gby065 29878211

[B29] Lisi M. P. Scattolin M. Fusaro M. Aglioti S. M. (2021). A Bayesian approach to reveal the key role of mask wearing in modulating projected interpersonal distance during the first COVID-19 outbreak. PLoS ONE 16:e0255598. 10.1371/journal.pone.0255598 34375361 PMC8354471

[B30] Lugli L. Iani C. Nicoletti R. Ricciardelli P. (2014). L'attivazione emotiva legata all'elaborazione delle espressioni: effetti di *priming affettivo* in bambini e adolescenti. Giornale Italiano di Psicologia, XLI, 229–238. 10.1421/77214

[B31] Maggi G. Baldassarre I. Barbaro A. Cavallo N. D. Cropano M. Nappo R.. (2021). Mental health status of Italian elderly subjects during and after quarantine for the COVID-19 pandemic: a cross-sectional and longitudinal study. Psychogeriatrics 21, 540–551. 10.1111/psyg.12703 33955115 PMC8242477

[B32] Mazzuca C. Falcinelli I. Michalland A.-H. Tummolini L. Borghi A. M. (2021). Differences and similarities in the conceptualization of COVID-19 and other diseases in the first Italian lockdown. Sci. Rep. 11, 18303. 10.1038/s41598-021-97805-3 34526599 PMC8443562

[B33] Meyer D. E. Schvaneveldt R. W. (1971). Facilitation in recognizing pairs of words: evidence of a dependence between retrieval operations. J. Exp. Psychol. 90, 227. 10.1037/h0031564 5134329

[B34] Montefinese M. Ambrosini E. Fairfield B. Mammarella N. (2014). The adaptation of the affective norms for English words (ANEW) for Italian. Behav. Res. Methods 46, 887–903. 10.3758/s13428-013-0405-3 24150921

[B35] Murphy S. T. Zajonc R. B. (1993). Affect, cognition, and awareness: affective priming with optimal and suboptimal stimulus exposures. J. Pers. Soc. Psychol. 64, 723. 10.1037/0022-3514.64.5.723 8505704

[B36] R Core Team (2016). R: A Language and Environment for Statistical Computing. Vienna: R Foundation for Statistical Computing. Available online at: https://www.R-project.org/ (accessed April 21, 2022).

[B37] Raftery A. E. (1999). Bayes factors and BIC. Sociol. Methods. Res. 27, 411–427. 10.1177/0049124199027003005

[B38] Rouder J. N. Speckman P. L. Sun D. Morey R. D. Iverson G. (2009). Bayesian *t* tests for accepting and rejecting the null hypothesis. Psychon. Bull. Rev. 16, 225–237. 10.3758/PBR.16.2.225 19293088

[B39] Santangelo G. Baldassarre I. Barbaro A. Cavallo N. D. Cropano M. Maggi G.. (2021). Subjective cognitive failures and their psychological correlates in a large Italian sample during quarantine/self-isolation for COVID-19. Neurol. Sci. 42, 2625–2635. 10.1007/s10072-021-05268-1 33914195 PMC8082482

[B40] Scerrati E. Baroni G. Borghi A. M. Galatolo R. Lugli L. Nicoletti R. (2015). The modality-switch effect: visually and aurally presented prime sentences activate our senses. Front. Psychol. 6:1668. 10.3389/fpsyg.2015.01668 26579049 PMC4627474

[B41] Scerrati E. Iani C. Rubichi S. (2021). “Does the activation of motor information affect semantic processing?,” in Concepts in Action, eds L. Bechberger, K.-U. Kühnberger, and M. Liu (Cham: Springer), 153–166. 10.1007/978-3-030-69823-2_7

[B42] Scerrati E. Lugli L. Nicoletti R. Borghi A. M. (2017). The multilevel modality-switch effect: what happens when we see the bees buzzing and hear the diamonds glistening. Psychon. Bull. Rev. 24, 798–803. 10.3758/s13423-016-1150-2 27542801

[B43] Scerrati E. Marzola G. Villani C. Lugli L. D'Ascenzo S. (2022). Nuovi scenari per gli esperimenti in psicologia: la modalità online. Giornale Italiano di Psicologia.

[B44] Spruyt A. Hermans D. Houwer J. D. Eelen P. (2002). On the nature of the affective priming effect: affective priming of naming responses. Soc. Cogn. 20, 227–256. 10.1521/soco.20.3.227.2110617533884

[B45] Spruyt A. Tibboel H. De Schryver M. De Houwer J. (2018). Automatic stimulus evaluation depends on goal relevance. Emotion 18, 332. 10.1037/emo0000361 29154588

[B46] Szczurek L. Monin B. Gross J. J. (2012). The stranger effect: the rejection of affective deviants. Psychol. Sci. 23, 1105–1111. 10.1177/0956797612445314 22961772

[B47] Tzavella L. Maizey L. Lawrence A. D. Chambers C. D. (2020). The affective priming paradigm as an indirect measure of food attitudes and related choice behaviour. Psychon. Bull. Rev. 27, 1397–1415. 10.3758/s13423-020-01764-1 32607847 PMC7704519

[B48] Villani C. D'Ascenzo S. Scerrati E. Ricciardelli P. Nicoletti R. Lugli L. Wearing the face mask affects our social attention over space [manuscript submitted for publication]. 10.3389/fpsyg.2022.923558 PMC9386249 35992481

[B49] Wagenmakers E. J. (2007). A practical solution to the pervasive problems of *p* values. Psychon. Bull. Rev. 14, 779–804. 10.3758/BF03194105 18087943

[B50] Ward J. H. Jr.. (1963). Hierarchical grouping to optimize an objective function. J. Am. Stat. Assoc. 58, 236–244. 10.1080/01621459.1963.10500845

[B51] Warriner A. B. Kuperman V. Brysbaert M. (2013). Norms of valence, arousal, and dominance for 13,915 English lemmas. Behav. Res. Methods 45, 1191–1207. 10.3758/s13428-012-0314-x 23404613

[B52] Wentura D. (2000). Dissociative affective and associative priming effects in the lexical decision task: yes versus no responses to word targets reveal evaluative judgment tendencies. J. Exp. Psychol. Learn. Mem. Cogn. 26, 456. 10.1037/0278-7393.26.2.456 10764106

[B53] Wu C. Zhang J. Yuan Z. (2021). Exploring affective priming effect of emotion-label words and emotion-laden words: an event-related potential study. Brain Sci. 11, 553. 10.3390/brainsci11050553 33925670 PMC8145978

[B54] Xie W. Campbell S. Zhang W. (2020). Working memory capacity predicts individual differences in social-distancing compliance during the COVID-19 pandemic in the United States. Proc. Natl. Acad. Sci.U. S. A. 117, 17667–17674. 10.1073/pnas.2008868117 32651280 PMC7395511

[B55] Xu P. Cheng J. (2021). Individual differences in social distancing and mask-wearing in the pandemic of COVID-19: the role of need for cognition, self-control and risk attitude. Pers. Individ. Dif. 175, 110706. 10.1016/j.paid.2021.110706 33551529 PMC7847399

